# Effects of Social Isolation on Mortality in Older Adults: Do Age and Gender Matter?

**DOI:** 10.1093/geroni/igaf122.3109

**Published:** 2025-12-31

**Authors:** Lydia Li, Jay Kayser, Kyungeun Song

**Affiliations:** University of Michigan, Ann Arbor, Michigan, United States; University of Michigan, Ann Arbor, Michigan, United States; University of Michigan, Ann Arbor, Michigan, United States

## Abstract

Social isolation is a recognized risk factor for adverse health outcomes, yet the magnitude of its effect on mortality and differences between genders remain unclear. This study examines whether social isolation predicts all-cause mortality and whether this relationship varies by gender. We analyzed data from the National Health and Aging Trends Study (NHATS), an ongoing nationally representative panel study of Medicare beneficiaries that began in 2011. The analytic sample included 7,186 community-living respondents at baseline, of whom a weighted 28.5% had died by 2023. Despite being a widely used construct, a gold standard to measure social isolation does not exist. Researchers have developed two NHATS-based measures (Pohl and Cudjoe). We used both in weighted Cox proportional-hazard models to test the main effect of social isolation and its interaction with gender, adjusting for sociodemographic, neighborhood, and health characteristics. Both measures of social isolation were significant predictors of mortality risk, though Pohl’s measure demonstrated a stronger effect than Cudjoe’s. Further, only Pohl’s measure had a statistically significant interaction effect with gender (F [2, 55] = 4.67, p =.01). Specifically, the risk of all-cause mortality was 56% and 2.1 times higher, respectively, for somewhat and very isolated men compared to not-isolated men. Among women, the risk of mortality was 33% higher for the very-isolated relative to the not-isolated. The findings suggest that the operationalization of social isolation matters. Overall, our findings show that social isolation hastens death in older adults, particularly for men. Targeted interventions to reduce social isolation may prevent premature mortality.

